# Persistent C-peptide secretion is associated with favourable CGM metrics in adults with type 1 diabetes

**DOI:** 10.1007/s00125-025-06578-1

**Published:** 2025-10-30

**Authors:** Roland H. Stimson, Anna R. Dover, Catriona Clarke, Carina Conceicao, Lindsay McDonald, Lucy Miller, Helen Wise, Shareen Forbes, Rohana J. Wright, Marcus J. Lyall, Mark W. J. Strachan, Fraser W. Gibb

**Affiliations:** 1 Edinburgh Centre for Endocrinology & Diabetes, NHS Lothian, Edinburgh, UK; 2 University/BHF Centre for Cardiovascular Science, Queen’s Medical Research Institute, University of Edinburgh, Edinburgh, UK; 3 Department of Clinical Biochemistry, NHS Lothian, Edinburgh, UK

**Keywords:** Clinical diabetes, Clinical science, Devices, Human, Hypoglycaemia

## Abstract

**Aims/hypothesis:**

Residual endogenous insulin secretion, reflected by measurable C-peptide, has been linked to improved glycaemic management in type 1 diabetes. We aimed to assess the relationship between random plasma C-peptide levels and continuous glucose monitoring (CGM) metrics in a large, real-world cohort of adults with type 1 diabetes.

**Methods:**

We conducted a cross-sectional analysis of adults with type 1 diabetes attending a single UK centre. Inclusion criteria were diabetes duration >1 year, random plasma glucose >4 mmol/l at C-peptide sampling and ≥70% data completeness on the Freestyle Libre 2 CGM device within the corresponding month. Associations between C-peptide categories (<50 pmol/l to >400 pmol/l) and CGM/HbA_1c_ outcomes were assessed using non-parametric tests and multivariable logistic regression.

**Results:**

In total, 945 adults with type 1 diabetes were included with a median age of 45 years (33–57) and median diabetes duration of 18 years (7–29). Of these, 54% were male and median HbA_1c_ was 63 mmol/mol (54–73) (7.9% [7.1–8.8]). Higher C-peptide levels were associated with shorter diabetes duration (OR 0.87 per year, *p*<0.001), older age at diagnosis (OR 1.04 per year, *p*<0.001) and male sex (OR 1.44, *p*=0.042). Higher C-peptide was significantly associated with favourable CGM metrics, including lower time below range (2% [1–5] in those with C-peptide <50 pmol/l vs 1% [0–3] in those with C-peptide 101–200 pmol/l), lower glucose variability (glucose CV 37.5% [34.2–42.0] in those with C-peptide <50 pmol/l vs 32.5% [29.0–36.6] in those with C-peptide 101–200 pmol/l), higher time in range (45.0% [32.0–61.0] in those with C-peptide <50 pmol/l vs 55.0% [37.0–66.5] in those with C-peptide 50–100 pmol/l) and favourable hyperglycaemia measures (time above 13.9 mmol/l 20.0% [9.0–36.0] in those with C-peptide <50 pmol/l vs 10.0% [5.5–31.5] in those with C-peptide 50–100 pmol/l) (*p*<0.05 for all pairwise comparisons). C-peptide ≥100 pmol/l was independently associated with meeting time below range <4% (OR 5.4, *p*<0.001), and C-peptide ≥100 pmol/l was also associated with achieving HbA_1c_ <53 mmol/mol (7%) (OR 1.8, *p*=0.043. No significant glycaemic differences were seen between individuals with C-peptide <10 pmol/l and 10–49 pmol/l.

**Conclusions/interpretation:**

Random C-peptide measurement in routine care identifies adults with type 1 diabetes who are more likely to achieve CGM and HbA_1c_ targets. Differences in glycaemic metrics are clinically meaningful at thresholds ≥100 pmol/l. These findings support efforts to preserve residual beta cell function and highlight the potential value of C-peptide in individualising therapy.

**Graphical Abstract:**

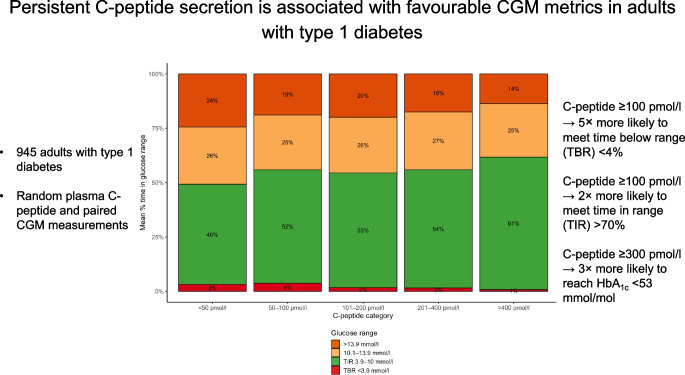

**Supplementary Information:**

The online version contains peer-reviewed but unedited supplementary material available at 10.1007/s00125-025-06578-1.


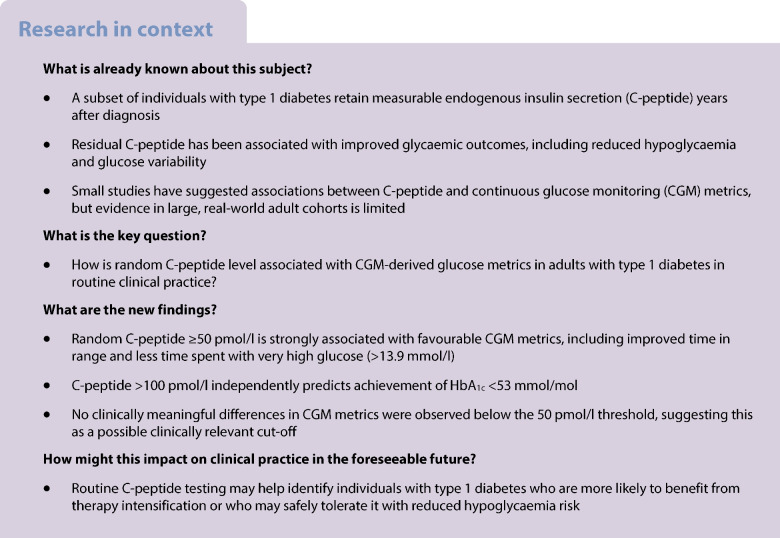


## Introduction

Type 1 diabetes is defined by autoimmune destruction of pancreatic beta cells, typically resulting in absolute insulin deficiency. However, a proportion of individuals with type 1 diabetes retain measurable endogenous insulin secretion, reflected by detectable C-peptide levels, several years after their initial diagnosis [1]. This residual beta cell function has been increasingly recognised as clinically meaningful, with several studies linking it to improved glycaemic outcomes and reduced risk of diabetes-related complications [2–4].

Our previous work demonstrated that persistent C-peptide secretion in adults with type 1 diabetes was associated with lower time below range (TBR) and reduced glucose variability, suggesting a protective role against hypoglycaemia and glycaemic excursions [3]. Since then, further evidence has strengthened the association between C-peptide and favourable glucose metrics. Notably, a 2023 study by Fuhri Snethlage et al reported that higher urinary C-peptide-to-creatinine ratios in people with type 1 diabetes were associated with longer time in range (TIR), shorter TBR and time above range (TAR), lower glucose CV and improved HbA_1c_ levels. Importantly, individuals with preserved beta cell function also required lower total daily insulin doses [4]. These findings reinforce the notion that residual insulin production contributes to more stable glucose management. This has implications for therapeutic approaches that maintain C-peptide persistence. In adults, those randomised to intensive therapy in the Diabetes Control and Complications Trial were found to have slower decline in C-peptide [5]. However, in children, randomisation to improved glycaemic management with automated insulin delivery did not have a significant impact upon C-peptide persistence [6]. Recently, disease-modifying therapies, which address the immune response in early type 1 diabetes, have shown efficacy in C-peptide preservation [7] and they may have promise in improving glycaemic management [8].

In this study, we performed a cross-sectional analysis in a well-characterised cohort of adults with type 1 diabetes to examine associations between residual C-peptide secretion and a comprehensive range of continuous glucose monitoring (CGM)-derived glucose metrics. By focusing on clinically relevant thresholds and real-world data we aimed to clarify the glycaemic significance of persistent endogenous insulin production.

## Methods

### Study design and participants

We carried out a cross-sectional study in 945 adults with type 1 diabetes who were using CGM (Freestyle Libre 2) and had available random plasma C-peptide results. The Edinburgh Centre for Endocrinology & Diabetes provides diabetes care to approximately 5000 adults with type 1 diabetes. Since July 2017, our centre has routinely measured random C-peptide levels in individuals with type 1 diabetes to identify potential misclassifications such as monogenic or type 2 diabetes (typically 3 years or more since onset of diabetes) [9].

For inclusion in this study, participants were required to have:

- Diabetes duration of over 1 year;
- Plasma glucose level >4 mmol/l at the time of C-peptide measurement;
- 2 weeks of CGM data available in the month corresponding to the C-peptide measurement (obtained from the LibreView portal);
- ≥70% CGM data completeness.

Since early 2018, the National Health Service-funded Freestyle Libre CGM device has been routinely available in our centre for people with diabetes using multiple daily insulin injections or continuous subcutaneous insulin infusion (CSII).

As this observational study was based entirely on routine clinical care and routinely collected data, ethics approval was not necessary.

### Outcomes

The key analysis assessed differences in CGM metrics by C-peptide category (<50 pmol/l, 50–100 pmol/l, 101–200 pmol/l, 201–400 pmol/l and >400 pmol/l). Categories were selected to reflect clinically relevant thresholds, assay detection limits, potential non-linear effects and, pragmatically, for reasonable numeric balance between groups. Detailed C-peptide values below the standard reporting threshold of 50 pmol/l were manually retrieved for a subset of individuals (346/602 individuals with C-peptide <50 pmol/l), allowing us to assess differences in CGM metrics between those with C-peptide <10 pmol/l and 10–49 pmol/l. Granular data were not available in all individuals with C-peptide <50 pmol/l as results reported for clinical use are reported as <50 pmol/l. Detailed results (further quantifying values <50 pmol/l) involved manual retrieval of data from laboratory analysers. Random plasma C-peptide was measured using a Roche immunoassay on the Cobas e411 platform. We assessed the full range of CGM metrics as described in the ‘Clinical Targets for Continuous Glucose Monitoring Data Interpretation’ consensus document [10]. HbA_1c_ recorded on the day of C-peptide measurement was available in 868/945 (92%). Clinical and demographic data were obtained from Scottish Care Information-Diabetes (SCI-Diabetes, the Scottish national diabetes register). Biological sex was determined from information recorded in SCI-Diabetes. Socioeconomic deprivation was assessed by the Scottish Index of Multiple Deprivation (SIMD; an area-based measure of relative deprivation, where quintile 1 is most deprived). Race/ethnicity data were not assessed as the clinic population was predominantly of White European background. Given the lack of diversity, meaningful comparisons by race/ethnicity could not be undertaken.

### Statistics

Data are presented as median (IQR) except for low glucose metrics which are presented as mean (SD). Unpaired data were compared with Wilcoxon rank-sum test. Comparison across multiple C-peptide categories was made with Kruskal–Wallis test and subsequent pairwise assessment with pairwise Wilcox test with Bonferroni correction. Categorical data were compared by χ^2^ test. Logistic regression analysis assessed independent predictors of those meeting CGM and HbA_1c_ targets.

To explore potential non-linear associations between C-peptide and glycaemic outcomes, we performed restricted cubic spline regression in individuals with quantified, non-zero values for C-peptide (*n*=392). Models were fitted with log-transformed C-peptide as the predictor and continuous CGM metrics as outcomes. Knots were placed at default quantiles. Significance of the overall and non-linear associations was assessed using ANOVA.

We considered *p* values <0.05 statistically significant. Statistical analyses were performed using R (version 4.5.0; R Foundation for Statistical Computing, Vienna, Austria; https://www.r-project.org/).

## Results

### Characteristics of cohort

**Fig. 1:**
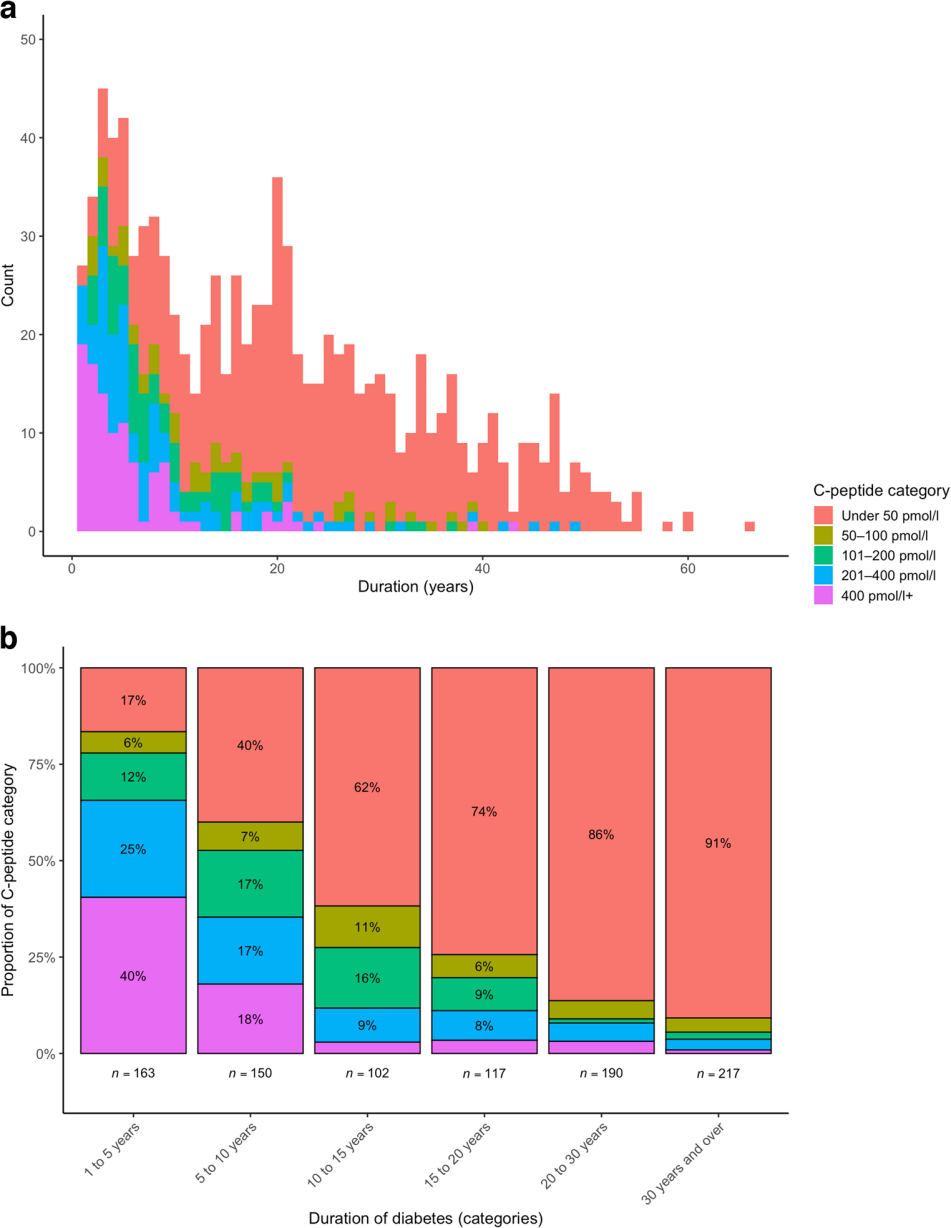
(**a**) Histogram of diabetes duration, stratified by C-peptide category. (**b**) Relationship between duration category and C-peptide category (*p*<0.001 for trend)

Cohort characteristics are presented in Table 1. Median age was 45 years (33–57) and median duration of diabetes was 18 years (7–29). In total, 54% were male and 19% were CSII users. Median HbA_1c_, at the time of C-peptide measurement, was 63 mmol/mol (54–73) (7.9% [7.1–8.8]). CGM data are presented in Table 2. Median mean glucose was 10.2 mmol/l (8.7–12.1), median TIR was 49% (34–67) and TBR was 1% (0–4). Lower C-peptide category was strongly associated with longer duration of diabetes (Fig. 1) and younger age at diagnosis of diabetes (Table 1). In logistic regression analysis (electronic supplementary material [ESM] Table 1), shorter diabetes duration (OR 0.87 per year, *p*<0.001), male sex (OR 1.44, *p*=0.042), older age (OR 1.04 per year, *p*<0.001) and current non-smoking status (OR 0.48 for current smokers, *p*=0.011) were all associated with a higher likelihood of persistent C-peptide secretion (≥50 pmol/l). Autoantibody results (GAD, IA-2 or ZnT8) were available in 82% with C-peptide >400 pmol/l and in 12% of those with C-peptide <50 pmol/l. At least one autoantibody was positive in 88% of those with C-peptide >400 pmol/l and in 82% of those with C-peptide <50 pmol/l (*p*=0.547).

**Table 1:** Clinical and demographic characteristics of the total cohort and stratified by C-peptide category

Characteristic	Total cohort (*n*=945)	C-peptide category					*p*
		<50 pmol/l (*n*=602)	50–100 pmol/l (*n*=55)	101–200 pmol/l (*n*=78)	201–400 pmol/l (*n*=101)	>400 pmol/l (*n*=109)	
Age (years)	45.4 (32.6–56.7)	45.6 (32.6–56.6)	48.9 (35.5–57.3)	40.2 (29.3–52.2)	45.4 (30.8–56.4)	46.6 (35.5–58.7)	<0.001
Age at diagnosis (years)	24.0 (13.0–36.0)	17.0 (10.0–28.0)	29.0 (19.5–41.5)	29.5 (21.0–39.5)	32.0 (23.8–46.0)	39.0 (30.0–52.0)	0.110
Duration (years)	17.7 (7.1–29.0)	23.3 (14.9–34.4)	12.9 (6.4–20.5)	7.6 (4.8–14.5)	6.4 (3.6–14.0)	4.0 (2.1–8.2)	<0.001
Sex (male)	53.5%	51.7%	56.4%	50.0%	56.4%	62.4%	0.268
BMI (kg/m^2^)	27.3 (24.2–31.3)	27.8 (24.2–31.9)	27.6 (23.9–30.5)	26.5 (24.0–30.5)	26.4 (23.9–29.9)	26.9 (24.4–30.9)	0.173
SIMD quintiles 1 & 2 (most deprived)	29.3%	30.2%	26.9%	24.7%	23.7%	34.7%	0.409
Current smoker	12.1%	13.0%	9.1%	11.5%	8.9%	12.1%	0.759
Ever smoker	37.1%	38.9%	32.7%	34.6%	28.7%	39.3%	0.317
CSII	18.8%	24.3%	18.2%	14.1%	7.9%	2.8%	<0.001
HbA_1c_ (mmol/mol)	63 (54–73)	63 (55–73)	63 (52–85)	63 (54–76)	62 (51–70)	58 (50–72)	0.011
HbA_1c_ (%)	7.9 (7.1–8.8)	7.9 (7.2–8.8)	7.9 (6.9–9.9)	7.9 (7.1–9.1)	7.8 (6.8–8.6)	7.4 (6.7–8.7)	0.011

Values are median (IQR) or percentage

*p* values refer to comparisons across all five C-peptide categories using Kruskal–Wallis or χ^2^ test, as appropriate

**Table 2:** Summary of CGM metrics for the total cohort and stratified by C-peptide category

Variable	Total cohort (*n*=945)	C-peptide category					*p*
		<50 pmol/l (*n*=602)	50–100 pmol/l (*n*=55)	101–200 pmol/l (*n*=78)	201–400 pmol/l (*n*=101)	>400 pmol/l (*n*=109)	
Mean glucose (mmol/l)	10.2 (8.7–12.1)	10.5 (9.0–12.3)	9.4 (8.5–11.6)	9.7 (8.7–11.1)	9.9 (7.9–11.6)*	9.0 (8.1–11.1)	<0.001
GMI (mmol/mol)	61 (54–70)	62 (55–71)	57 (53–68)	59 (54–65)	59 (50–67)*	55 (51–65)	<0.001
GMI (%)	7.7 (7.1–8.6)	7.8 (7.2–8.6)	7.4 (7.0–8.4)	7.5 (7.1–8.1)	7.5 (6.7–8.3)	7.2 (6.8–8.1)	<0.001
Sensor active (%)	94.0 (88.0–97.0)	94.0 (87.0–97.0)	93.0 (89.5–98.0)	95.5 (87.5–98.0)	95.0 (88.0–97.0)	95.0 (89.0–98.0)	0.291
Glucose >13.9 mmol/l	16.0 (6.0–33.0)	20.0 (9.0–36.0)	10.0 (5.5–31.5)*	12.5 (6.0–27.0)	11.0 (3.0–24.0)	4.0 (0.0–17.0)	<0.001
Glucose 10.1–13.9 mmol/l (%)	26.0 (20.0–33.0)	26.0 (21.3–31.8)	25.0 (21.0–30.5)	26.5 (18.5–33.8)	30.0 (16.0–37.0)	25.0 (16.0–35.0)	0.639
TAR (%)	48.0 (30.0–65.0)	51.0 (34.0–66.0)	40.0 (28.0–61.0)	42.5 (28.3–58.0)	45.0 (18.0–65.0)	33.0 (17.0–61.0)*	<0.01
TIR (%)	49.0 (34.0–67.0)	45.0 (32.0–61.0)	55.0 (37.0–66.5)*	53.0 (39.0–69.0)	55.0 (34.0–78.0)	67.0 (39.0–83.0)	<0.001
TBR (%)	1.0 (0.0–4.0)	2.0 (1.0–5.0)	2.0 (1.0–5.0)	1.0 (0.0–3.0)*	1.0 (0.0–2.0)	0.0 (0.0–1.0)	<0.001
Glucose 3.0–3.8 mmol/l (%)	0.0 (0.0–3.0) 1.6 (2.5)	1.0 (0.0–3.0) 2.0 (2.7)	1.0 (0.0–3.0) 2.6 (3.6)	1.0 (0.0–2.0) 1.4 (1.9)	0.0 (0.0–1.0) 0.8 (1.4)*	0.0 (0.0–0.0) 0.6 (1.6)	<0.001
Glucose <3.0 mmol/l (%)	0.0 (0.0–1.0) 0.4 (1.2)	0.0 (0.0–1.0) 0.6 (1.4)	0.0 (0.0–1.0) 0.7 (1.8)	0.0 (0.0–0.0) 0.2 (0.5)*	0.0 (0.0–0.0) 0.2 (0.5)	0.0 (0.0–0.0) 0.1 (0.3)	<0.001
CV glucose (%)	35.6 (30.8–40.0)	37.5 (34.2–42.0)	36.5 (33.1–40.0)	32.5 (29.0–36.6)*	31.0 (27.2–35.5)	26.9 (23.8–30.0)	<0.001
SD glucose (mmol/l)	3.7 (3.0–4.6)	4.0 (3.3–4.9)	3.5 (3.1–4.4)*	3.3 (2.8–4.0)	3.1 (2.6–3.7)	2.5 (1.9–3.1)	<0.001

All values are median (IQR) unless otherwise stated. Glucose <3.0 mmol/l and 3.0–3.8 mmol/l values are also presented as mean (SD) to provide more useful distributional data, as median and IQR were frequently zero

TIR refers to 3.9–10.0 mmol/l and TBR refers to <3.9 mmol/l

^*^The first C-peptide category for which pairwise comparison with the <50 pmol/l group reached statistical significance (*p*<0.05 after Bonferroni correction)

GMI, glucose management indicator

### Associations between C-peptide category and glucose metrics

**Fig. 2:**
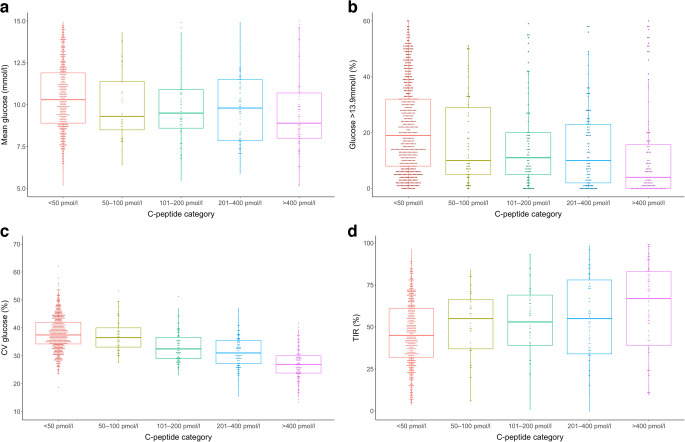
Selected comparison of mean glucose (**a**), glucose >13.9 mmol/l (**b**), CV glucose (**c**) and TIR (3.9–10.0 mmol/l) (**d**), with box plots of CGM metrics stratified by C-peptide category. Boxes represent the IQR, with horizontal lines showing the median. Whiskers extend to the most extreme values within 1.5 × IQR of the box. *p* values for trend across categories <0.001 for all panels shown (Kruskal–Wallis test)

Associations between C-peptide categories and CGM metrics are presented in Table 2. Increasing C-peptide concentration was associated with superior CGM metrics with respect to mean glucose, high glucose, low glucose and glucose variability, with some differences occurring between <50 pmol/l and 50–100 pmol/l (TIR and time with glucose >13.9 mmol/l) (Table 2). Selected comparisons of CGM metrics across C-peptide categories are presented in Fig. 2. HbA_1c_ was significantly lower in those with C-peptide >400 pmol/l with reference to C-peptide <50 pmol/l (63 mmol/mol [55–73] vs 58 mmol/mol [50–72], *p*=0.006) (7.9% [7.2–8.8] vs 7.5% [6.7–8.7]). Other detectable C-peptide groups were not associated with significantly lower HbA_1c_ compared with those with C-peptide <50 pmol/l.

In a subgroup of individuals where more granular C-peptide data were available, there was no significant difference in any CGM metric when comparing those with C-peptide <10 pmol/l (*n*=302) and those with C-peptide 10–49 pmol/l (*n*=44).

In those with detectable, detailed C-peptide levels (*n*=392), restricted cubic spline analysis demonstrated significant associations between log-transformed C-peptide and all CGM metrics assessed (ESM Figs 1–4). The relationships with TIR, TBR and glucose >13.9 mmol/l were broadly linear, while a significant non-linear association was observed for CV glucose, suggesting potential threshold effects at modest C-peptide levels (~50 pmol/l).

### Predictors of meeting glycaemic targets

**Fig. 3:**
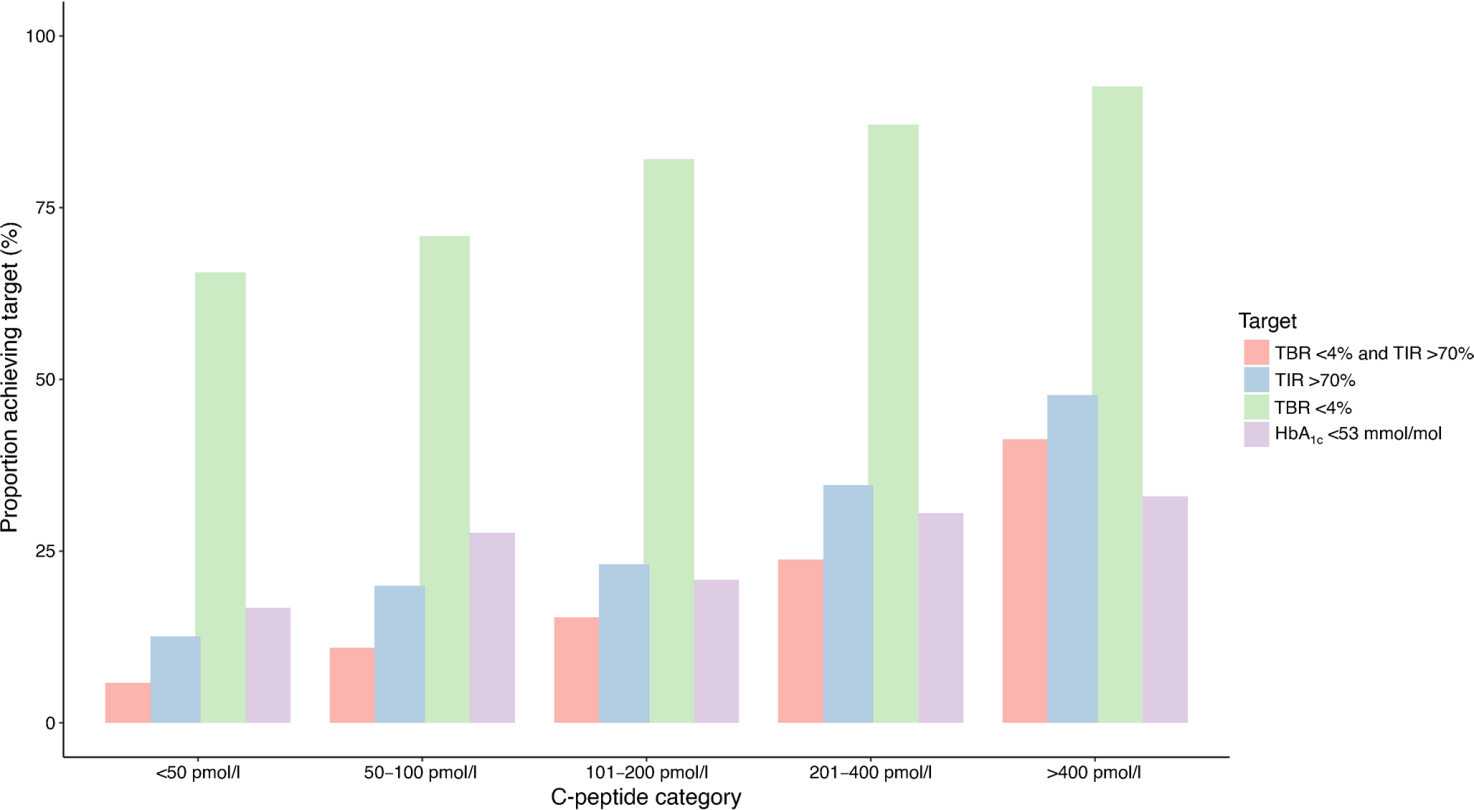
Proportion of individuals meeting CGM metric and HbA_1c_ targets. Targets are defined as TBR <4%, TIR >70% and HbA_1c_ <53 mmol/mol. *p*<0.001 for all comparisons (χ^2^ test for trend)

The proportion of individuals meeting CGM and HbA_1c_ targets by C-peptide category are presented in Fig. 3. In logistic regression analysis (ESM Table 2), with reference to C-peptide <100 pmol/l, C-peptide 100–300 pmol/l (OR 5.4, *p*<0.001) and C-peptide >300 pmol/l (OR 7.8, *p*<0.001) were associated with meeting the TBR <4% target. Higher mean glucose (OR 1.17 per mmol/l, *p*<0.001) and older age (OR 1.03 per year, *p*<0.001) were also associated with meeting the TBR target. With respect to the >70% TIR target, C-peptide 100–300 pmol/l (OR 2.2, *p*=0.002) and C-peptide >300 pmol/l (OR 4.8, *p*<0.001) were associated with meeting the target compared with those with C-peptide <100 pmol/l (ESM Table 3). Cigarette smokers (OR 0.34, *p*<0.001) and those with higher BMI (OR 0.95 per kg/m^2^, *p*<0.001) were less likely to achieve the TIR target. With reference to C-peptide <100 pmol/l, C-peptide 100–300 pmol/l (OR 1.8, *p*=0.043) and C-peptide >300 pmol/l (OR 3.0, *p*<0.001) were associated with a greater likelihood of HbA_1c_ <53 mmol/mol (7.0%) (ESM Table 4). CSII use was associated with a higher likelihood of HbA_1c_ <53 mmol/mol (7.0%) (OR 1.6, *p*=0.040) whereas cigarette smoking was associated with a lower likelihood (OR 0.19, *p *< 0.001).

## Discussion

We have shown that random C-peptide measurement, assessed during routine diabetes clinic appointments, is a robust predictor of favourable CGM metrics across all domains. Associations with very high glucose and TIR were observed with C-peptide over 50 pmol/l. Approximately 36% of our cohort had C-peptide >50 pmol/l and these were typically people with shorter duration of diabetes. As expected, rates of C-peptide persistence were substantially lower in those with longer duration of diabetes.

This is, to our knowledge, the largest assessment of associations between C-peptide and CGM metrics in adults with type 1 diabetes. The findings of favourable low glucose and variability metrics are consistent with our earlier published cohort study [3]. We did not observe associations with other glucose metrics in our earlier report but have in this larger cohort with higher HbA_1c_. Specifically, associations with TIR and high glucose metrics accord with a recently published cohort study in 489 adults with type 1 diabetes [4]. We have found that relatively modest C-peptide levels (50–100 pmol/l) were associated with greater TIR and lower rates of extreme hyperglycaemia (>13.9 mmol/l). The magnitude of difference across all CGM metrics is clinically meaningful and suggests that strategies to preserve endogenous insulin secretion are potentially important. The absence of any significant difference in key CGM metrics between those with C-peptide <10 pmol/l and 10–49 pmol/l suggests the clinically relevant threshold for C-peptide is around 50 pmol/l. C-peptide >50 pmol/l is where significant differences in SD glucose, TIR and extreme hyperglycaemia were observed.

Persistent C-peptide secretion was more likely in men than in women in logistic regression analysis. This accords with findings we have previously published [11] and with data from the Scottish type 1 bioresource cohort [12]. We found current cigarette smoking to be associated with a higher likelihood of undetectable C-peptide (<50 pmol/l), which we have also previously reported [11]. There is little in the current literature to confirm this finding or provide an explanatory mechanism. A small study in non-diabetic individuals suggested impaired insulin secretion in smokers [13] and a murine model of cigarette smoke exposure caused lower beta cell mass and beta cell dysfunction, ameliorated by antioxidant therapy [14].

C-peptide >100 pmol/l was independently associated with achieving the TBR <4% target and the TIR >70% target. C-peptide >300 pmol/l was associated with a greater likelihood of achieving an HbA_1c_ <53 mmol/mol. We have previously shown that cigarette smoking is associated with a lower likelihood of achieving HbA_1c_ targets [11]. This was confirmed in the current study and those findings extended to a lower likelihood of achieving TIR. Smoking is associated with greater insulin resistance [15] and this may also explain why higher BMI was associated with a lower likelihood of meeting TIR and HbA_1c_ targets. Male sex was an independent predictor of meeting HbA_1c_ targets despite not being associated with TIR or TBR targets. HbA_1c_ has been shown to be higher in women than in men despite equivalent sensor mean glucose [16] and this was also true of this cohort (data not shown). A small difference in HbA_1c_–glucose discordance at the margins of the HbA_1c_ threshold may have had a significant distorting effect. The relatively weak association between C-peptide and HbA_1c_ compared with stronger associations with CGM glucose metrics (including mean glucose and glucose >13.9 mmol/l), as well as the sex difference, hint towards the superiority of CGM-derived metrics over HbA_1c_ in assessing glucose exposure.

As this is an observational study, we cannot infer causation between persistent C-peptide and superior CGM metrics. However, it is unlikely that preserved beta cell function is a consequence of improved glycaemic management. Recent studies (albeit in children) have refuted the hypothesis that intensive glycaemic management preserves endogenous insulin secretion [6, 17]. Further support is derived from the absence of any significant association with HbA_1c_ and C-peptide status in our cohort in logistic regression analysis. The use of random C-peptide is not regarded as the gold standard for assessment of endogenous insulin secretion; however, it has the advantage of being practical for routine clinical use and has been shown to perform well in relation to mixed meal tolerance tests [18]. If anything, the use of random C-peptide assessment would underestimate relationships with CGM metrics rather than introduce false positive associations. Including those with high C-peptide concentration raises the possibility that the cohort contains individuals misclassified as having type 1 diabetes. However, most people with higher C-peptide were within 5 years of diagnosis, had no significant difference in BMI and had the same rates of antibody positivity as those with undetectable C-peptide. Furthermore, our centre has been systematically assessing for misclassification of diabetes since 2017 [9] and classification errors are not likely to be common within this cohort. This large cohort was established from routine clinical practice and is likely to be generalisable. The only biases affecting inclusion were whether individuals shared CGM data with the clinic and whether ≥70% CGM data capture was present. Median HbA_1c_ in the cohort (62.5 mmol/mol) was similar to the current median in our entire clinic population (63 mmol/mol), suggesting a reasonably representative sample. Although larger cohort size may explain the differences observed in this study compared with our smaller cohort from 2019 (which showed only associations with low glucose and variability metrics), it is possible that the use of a different C-peptide assay may be relevant. It is well recognised that results from the Roche C-peptide assay report significantly higher values than those obtained from the Abbott assay [19]; improved standardisation between assays is required to improve the generalisability of clinical research involving C-peptide. All CGM data were obtained from the Freestyle Libre 2 sensor system, which is likely to limit bias introduced by the variability in metrics obtained across different CGM systems [20]. Our cohort did not include people using automated insulin delivery which, intuitively, would have an impact across multiple CGM metrics, independent of C-peptide status.

In conclusion, persistent C-peptide secretion in adults with type 1 diabetes is associated with clinically meaningful differences in key CGM metrics and higher levels of achieving glycaemic targets. Therapies that durably preserve beta cell function are likely to have beneficial effects. Intensification of therapy may be possible with lower risk of hypoglycaemia in people with preserved C-peptide. Conversely, people with undetectable C-peptide may be at greater risk of dysglycaemia, and consideration should be given to priority referral for automated insulin delivery.

## Supplementary Information

Below is the link to the electronic supplementary material.

ESM (PDF 425 KB)

## Data Availability

The data that support the findings of this study are not publicly available due to the inclusion of sensitive patient information and restrictions related to NHS data governance and confidentiality policies. Requests for access to the data may be considered on a case-by-case basis and subject to appropriate ethical approvals and data-sharing agreements.

## Abbreviations

CGM: Continuous glucose monitoring
CSII: Continuous subcutaneous insulin infusion
SIMD: Scottish Index of Multiple Deprivation
TAR: Time above range
TBR: Time below range
TIR: Time in range
