# Supplementary material for: Persistent C-peptide secretion is associated with favourable CGM metrics in adults with type 1 diabetes
Source: Diabetologia. 2025 Oct 30;69(1):59–68. doi: 10.1007/s00125-025-06578-1 (PMC12686044; doi:10.1007/s00125-025-06578-1)

Electronic supplementary material

ESM table 1: Logistic regression predicting persistent C-peptide (>50pmol/l)

**Variable**	**Odds Ratio**	**95% CI**	**p-value**
Male (vs. Female)	1.44	1.01 – 2.06	0.042
Duration (years)	0.87	0.85 – 0.89	<0.001
Current Smoker	0.48	0.27 – 0.84	0.011
Age (years)	1.04	1.03 – 1.05	<0.001
HbA_{1c} (mmol/mol)	0.99	0.98 – 1.00	0.123

ESM table 2: Logistic Regression Predicting Achievement of TBR <4% Consensus Target

**Variable**	**Odds Ratio**	**95% CI**	**p-value**
Mean Glucose (mM)	1.77	1.59 – 1.98	<0.0001
C-peptide 100–300 pmol/l	5.37	2.83 – 10.17	<0.0001
C-peptide >300 pmol/l	7.78	3.82 – 15.82	<0.0001
Current Smoker	0.59	0.33 – 1.05	0.065
Diabetes Duration (years)	0.98	0.96 – 1.00	0.051
CSII Use	1.28	0.83 – 1.98	0.255
SIMD 1–2 (most deprived)	0.71	0.49 – 1.03	0.080
Age (years)	1.03	1.01 – 1.05	0.0002

ESM table 3: Logistic Regression Predicting Achievement of TIR >70% Consensus Target

Variable	Odds Ratio	95% CI	p-value
C-peptide 100–300 pmol/l	2.23	1.35 – 3.69	0.0018
C-peptide >300 pmol/l	4.76	2.83 – 8.01	<0.0001
Current Smoker	0.34	0.17 – 0.67	0.0020
SIMD 1–2 (most deprived)	0.76	0.51 – 1.13	0.176
BMI (kg/m²)	0.94	0.91 – 0.97	0.0005
Diabetes Duration (years)	1.00	0.98 – 1.02	0.815
CSII Use	1.25	0.79 – 1.96	0.343
Age (years)	1.01	0.99 – 1.02	0.280

ESM table 4: Logistic Regression Predicting Achievement of HbA_{1c} <53 mmol/mol (<7.0%)

Predictor	Odds Ratio	95%	p-value
BMI (per unit increase)	0.95	0.92 – 0.98	0.0034
Male sex	2.05	1.44 – 2.91	<0.0001
C-peptide 100–300 pmol/l	1.75	1.02 – 2.99	0.0426
C-peptide >300 pmol/l	3.04	1.75 – 5.30	<0.0001
Current smoker	0.19	0.08 – 0.46	<0.0002
Duration of diabetes (years)	1.01	0.99 – 1.03	0.1120
CSII use	1.60	1.02 – 2.52	0.0404
SIMD 1- 2 (most deprived)	0.78	0.51 – 1.21	0.2442
Age (years)	0.99	0.98 – 1.00	0.0870

ESM Figure 1.

Restricted cubic spline showing the association between log-transformed C-peptide and TIR.

The spline demonstrates a significant overall association between log C-peptide and TIR (P <0.001), consistent with a linear relationship (non-linearity P = 0.687). Shaded area represents 95% confidence intervals.

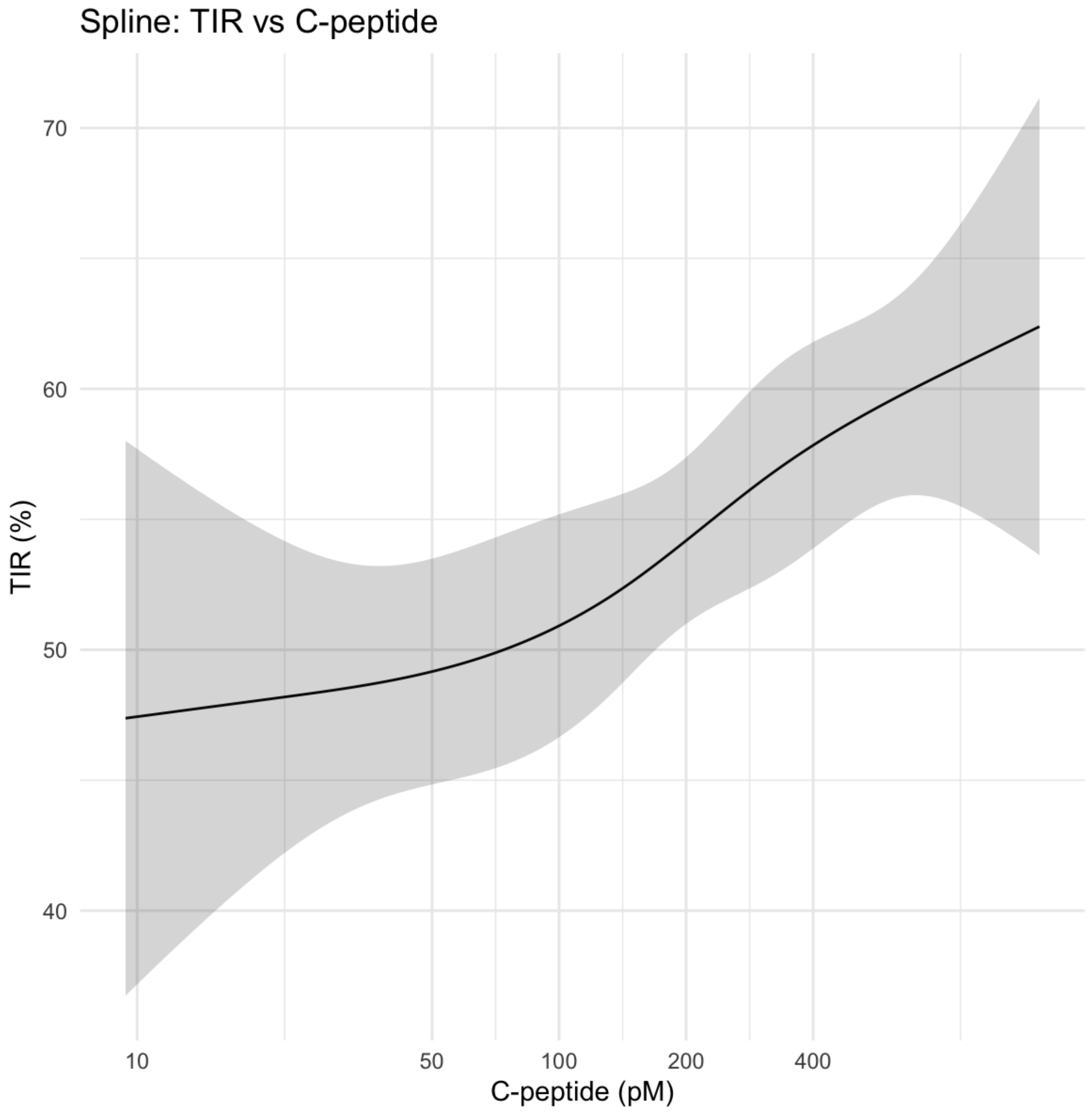

ESM Figure 2.

Restricted cubic spline showing the association between log-transformed C-peptide and TBR.

Log C-peptide is strongly associated with lower TBR ($P < 0.001$), with no evidence of non-linearity ($P = 0.275$), suggesting a consistent inverse relationship across the range of C-peptide values.

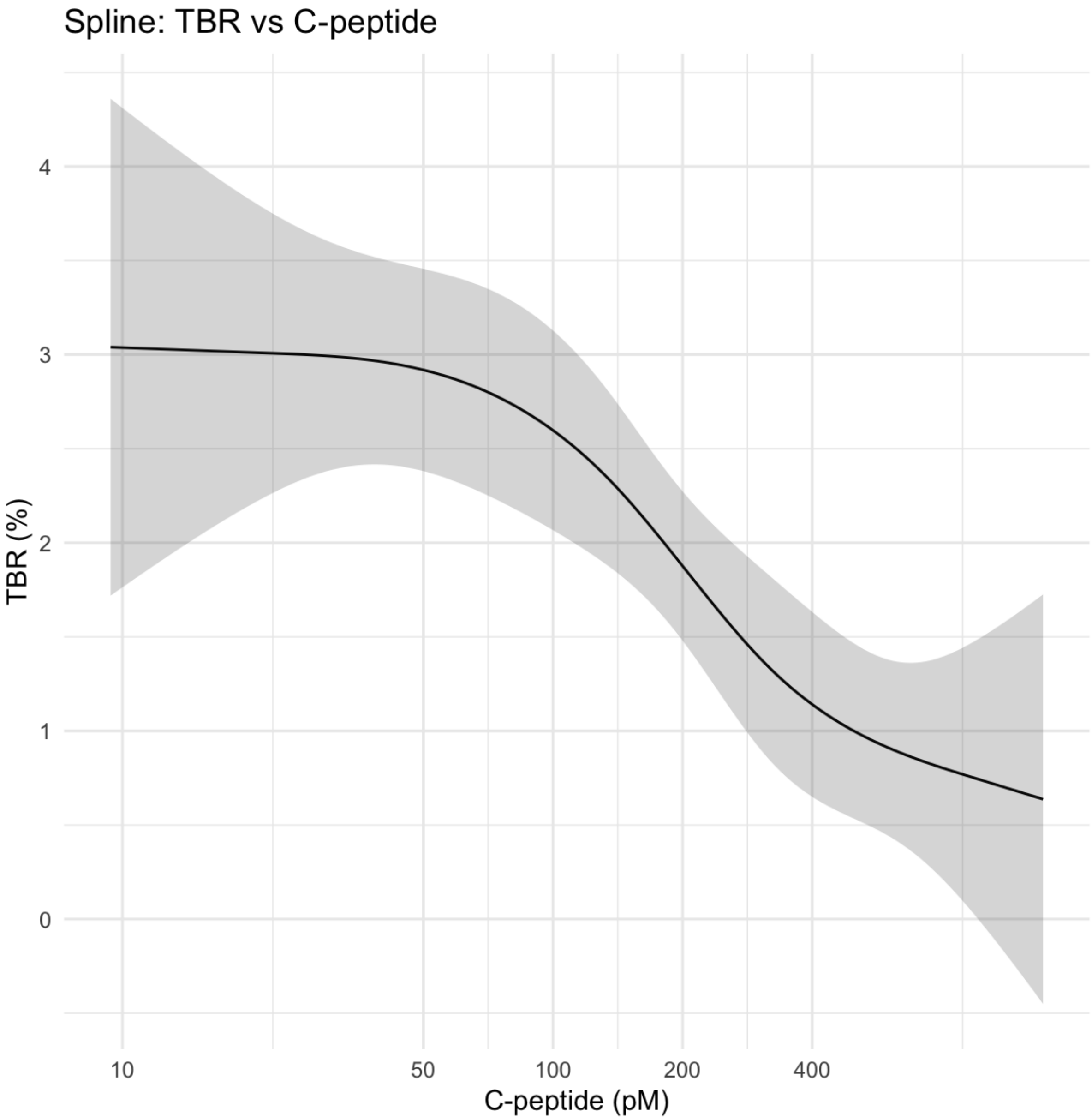

ESM Figure 3.

Restricted cubic spline showing the association between log-transformed C-peptide and glucose coefficient of variation (CV).

A significant non-linear relationship is observed between C-peptide and glucose variability (P < 0.001; non-linearity P = 0.004), suggesting that reductions in variability may become more pronounced above certain C-peptide thresholds.

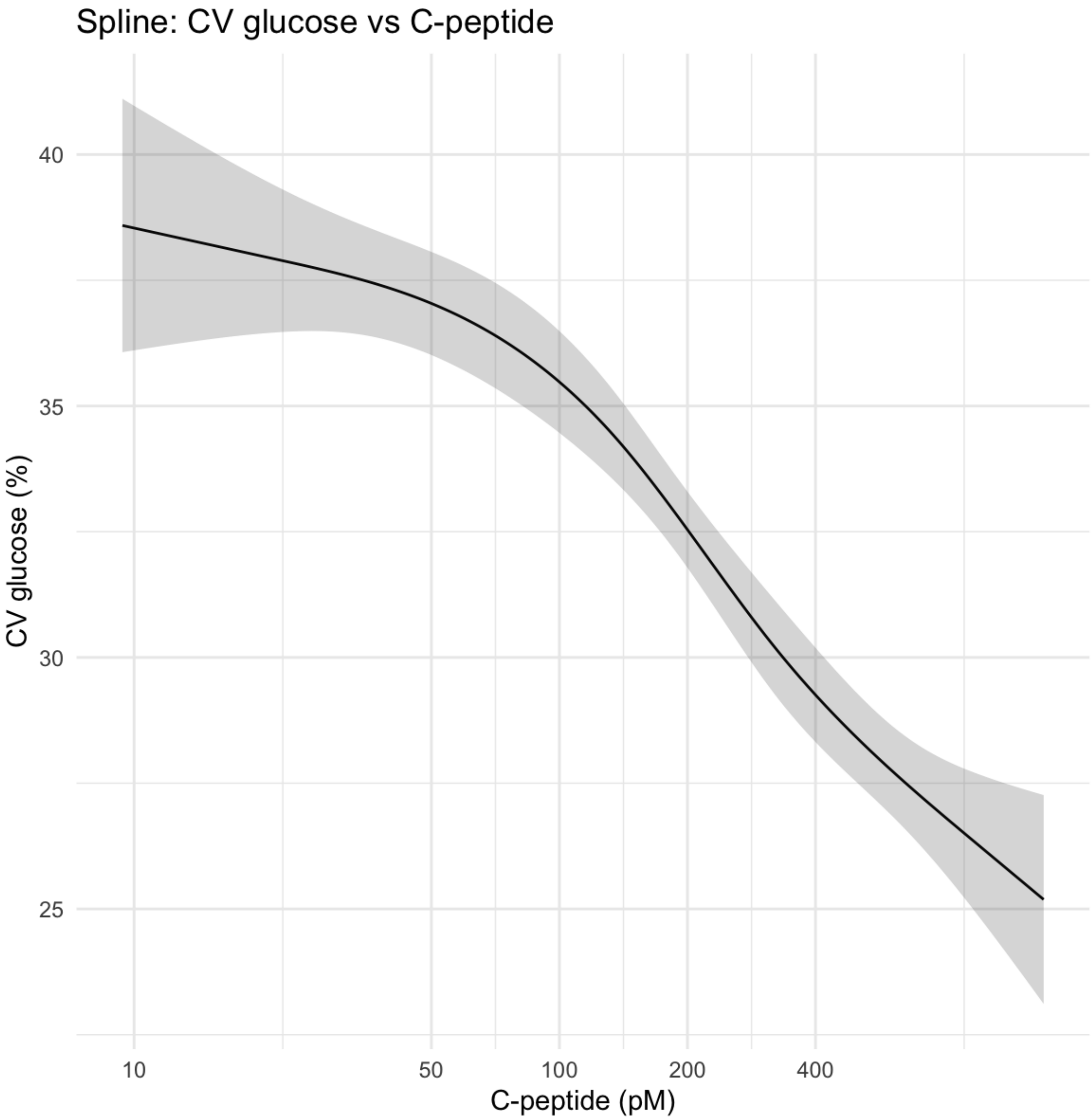

ESM Figure 4.

Restricted cubic spline showing the association between log-transformed C-peptide and time spent in very high glucose (>13.9 mmol/L).

Log C-peptide was modestly associated with reduced time in very high glucose (P = 0.035), with no significant non-linearity (P = 0.494), indicating a broadly linear trend.

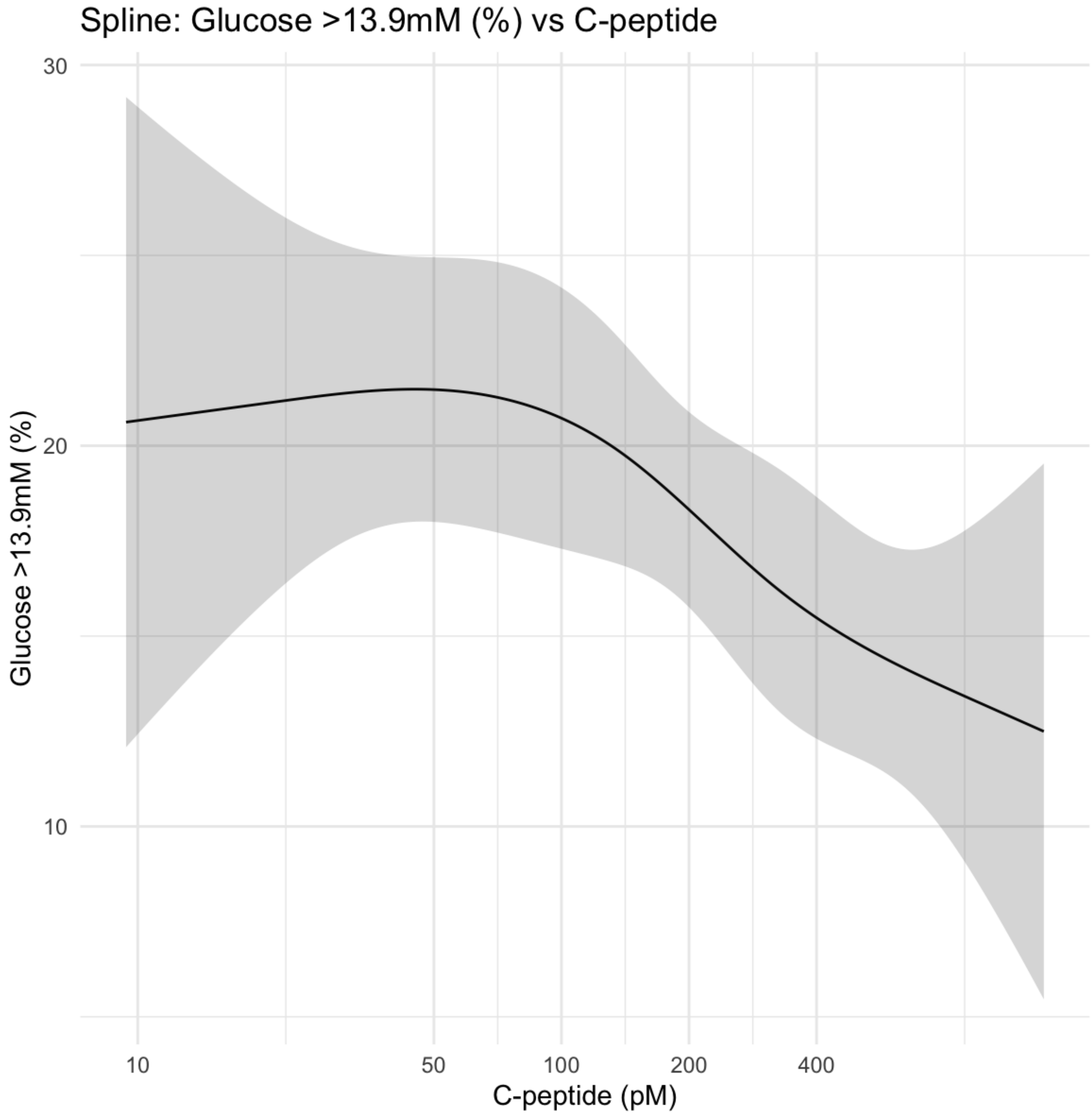

Supplement: Supplementary file 1 — ESM (PDF 425 KB) [file 125_2025_6578_MOESM1_ESM.pdf]
